# *Plasmodium vivax* in the Era of the Shrinking *P. falciparum* Map

**DOI:** 10.1016/j.pt.2020.03.009

**Published:** 2020-04-22

**Authors:** Ric N. Price, Robert J. Commons, Katherine E. Battle, Kamala Thriemer, Kamini Mendis

**Affiliations:** 1 Global Health Division, Menzies School of Health Research and Charles Darwin University, Darwin, Northern Territory, Australia; 2 Centre for Tropical Medicine and Global Health, Nuffield Department of Clinical Medicine, University of Oxford, Oxford, UK; 3 Mahidol-Oxford Tropical Medicine Research Unit (MORU), Faculty of Tropical Medicine, Mahidol University, Bangkok, Thailand; 4 Internal Medicine Services, Ballarat Health Services, Ballarat, Victoria, Australia; 5 Institute for Disease Modeling, Bellevue, WA, USA; 6 Faculty of Medicine, University of Colombo, Kynsey Road, Sri Lanka

## Abstract

*Plasmodium vivax* is an important cause of malaria, associated with a significant public health burden. Whilst enhanced malaria-control activities have successfully reduced the incidence of *Plasmodium falciparum* malaria in many areas, there has been a consistent increase in the proportion of malaria due to *P. vivax* in regions where both parasites coexist. This article reviews the epidemiology and biology of *P. vivax*, how the parasite differs from *P. falciparum*, and the key features that render it more difficult to control and eliminate. Since transmission of the parasite is driven largely by relapses from dormant liver stages, its timely elimination will require widespread access to safe and effective radical cure.

## Extent and Burden of *Plasmodium vivax*

**Figure 1:**
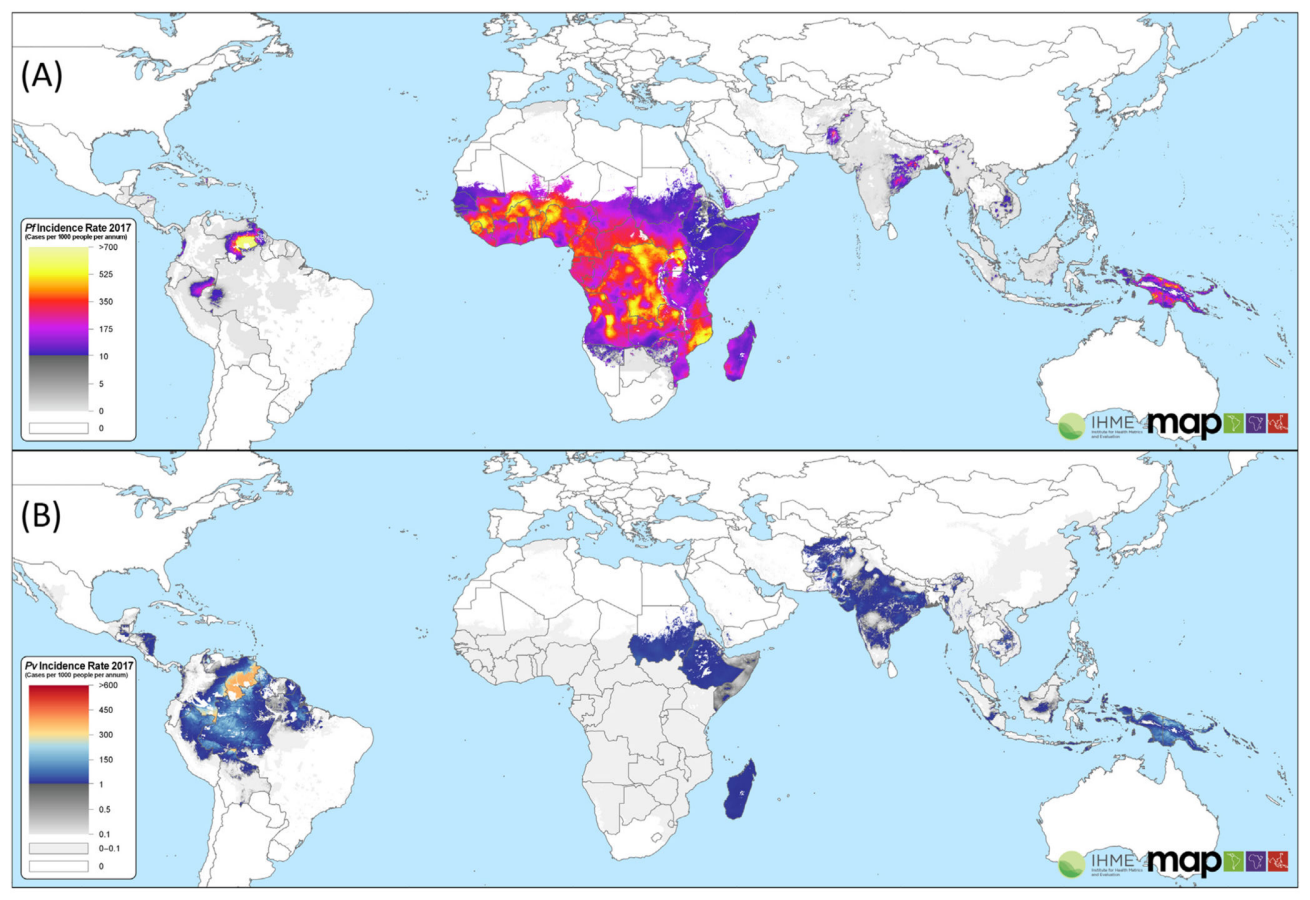
The Incidence of *Plasmodium falciparum* and *Plasmodium vivax* Malaria in 2017. *P. falciparum* (A) and *P. vivax* (B) data from [1,77] with permission.

**Figure 2:**
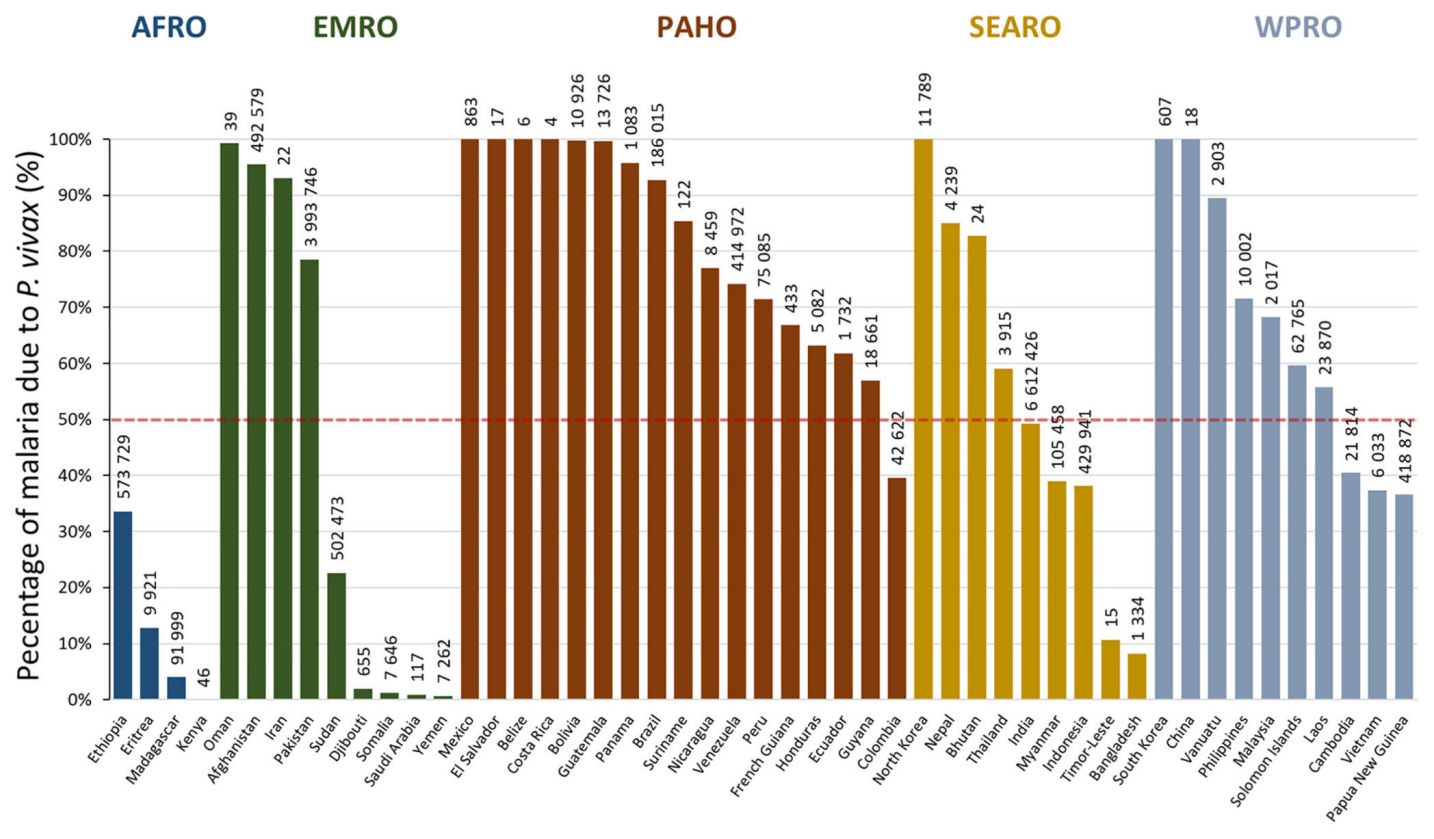
Proportion of Malaria Due to *Plasmodium vivax* by Country Grouped by World Health Organization (WHO) Regional Office. Numbers above columns represent the mean estimated cases in each country in 2017. Data extracted from post hoc estimates from [1] with permission, and available at https://malariaatlas.org/trends/region. Abbreviations: AFRO, Africa regional office; EMRO, Eastern Mediterranean regional office; PAHO, Pan American Health Organization; SEARO, South East Asia regional office; WPRO, West Pacific regional office.

**Figure 3:**
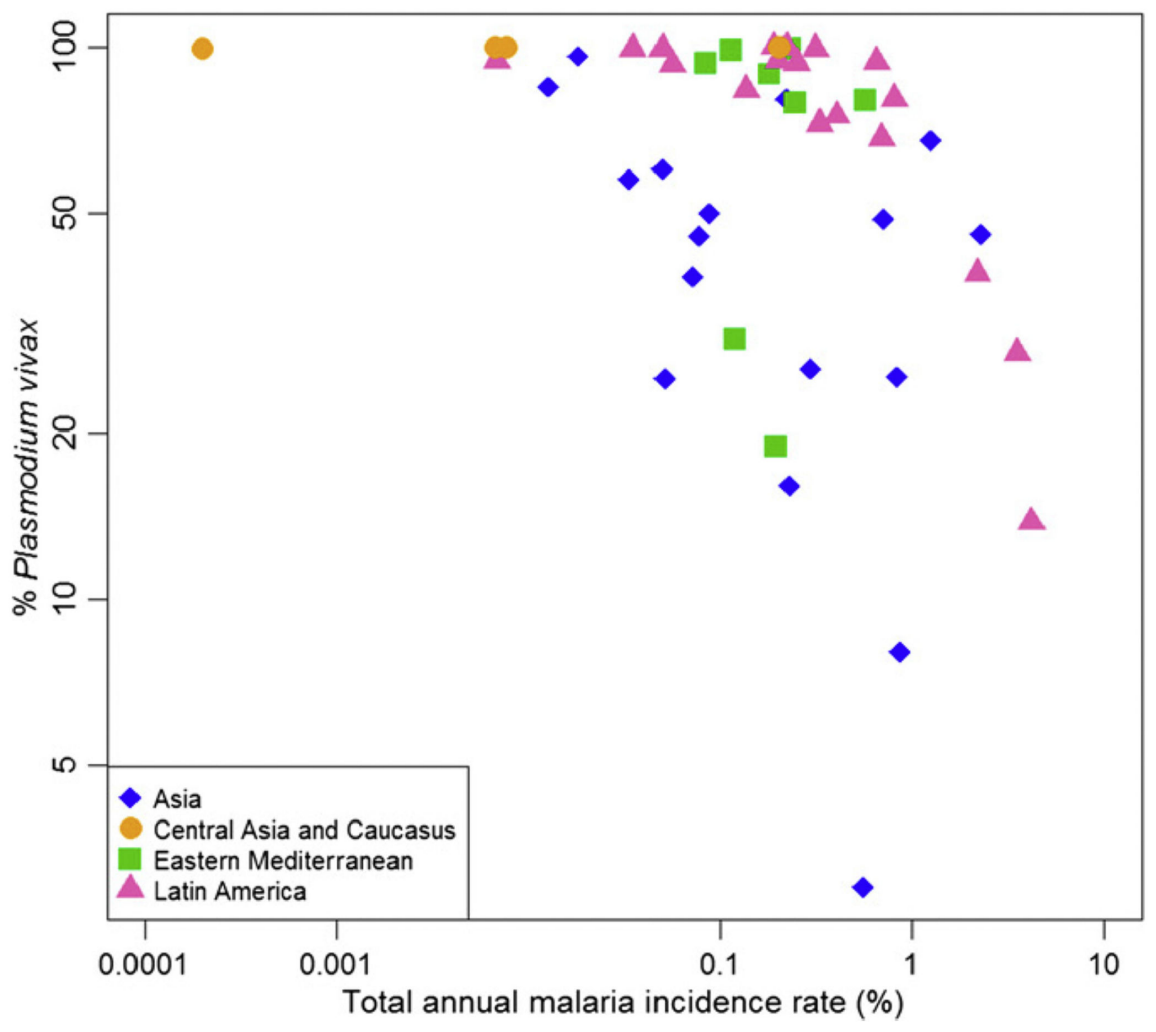
Variation in the Proportion of Malaria Cases Due to *Plasmodium vivax* Compared with the Annual Incidence of Malaria. The proportion of malaria due to *P. vivax* is highest in areas with a low malaria prevalence. Figure extracted, with permission, from [2]. The data points are colour-coded and shaped by region. The percentage of *P. vivax* for each country is derived from cases reported by the countries to the World Health Organization.

Of the five *Plasmodium* species that cause human malaria*, Plasmodium vivax* is the most geographically widespread. The parasite is capable of surviving quiescent for prolonged periods when conditions are not conducive to its ongoing transmission. A century ago *P. vivax* was prevalent in almost all countries; even though the vivax endemic world has shrunk considerably, over four billion people remain at risk of infection [1]. In 2017, transmission was reported from 49 countries across Central and South America, the Horn of Africa, Asia, and the Pacific islands (Figure 1). In almost two-thirds of coendemic countries *P. vivax* is the predominant species of malaria (Figure 2), the proportion of malaria attributable to the parasite being greatest in areas where the prevalence of malaria is low (Figure 3) [2,3].

Until recently the global burden of *P. vivax* malaria was derived from estimates of *P. falciparum*, the most prevalent species causing human malaria. However, a growing awareness of the public health importance of *P. vivax* has led to the strengthening of surveillance systems and better reporting practices of all of the *Plasmodium* species. The World Health Organization (WHO) first included *P. vivax* case estimates in its *World Malaria Report* (WMR) in 2013, documenting between 11.9 and 22 million *P. vivax* clinical cases per year [4]. Recent estimates, incorporating national surveillance data, prevalence surveys, and geospatial mapping, have revised the global burden to between 13.7 and 15 million cases in 2017 [1]. An estimated 82% (11.7 million cases) of the global vivax burden comes from four high-burden countries: India, Pakistan, Ethiopia, and Sudan.

In sub-Saharan Africa, where the burden of malaria is overwhelmingly attributable to *P. falciparum* (Figure 1), the low prevalence of *P. vivax* is attributed to a high proportion of the population having a Duffy-negative blood group. The Duffy antigen is an important molecule for the erythrocytic invasion of *P. vivax*, and the lack of the receptor on red blood cells reduces the risk of infection [5]. However, a recent review of clinical and vector data has shown that *P. vivax* is present across almost all malaria-endemic regions of Africa [6].

## Biological Differences between *P. falciparum* and *P. vivax*

The control and elimination of *P. vivax* is more challenging than that of *P. falciparum*, a reflection of key differences in parasite and vector biology. *P. vivax* usually circulates at low peripheral parasite densities, which – whilst still transmissible to the mosquito vector – creates significant challenges for diagnosing infected individuals. Furthermore, rapid diagnostic tests (RDTs) for *P. vivax* have reduced sensitivity compared with those used to diagnose *P. falciparum* [7]. Rapid identification of vivax malaria and interruption of transmission is further complicated by recurrent infections early in life, resulting in faster acquisition of immunity than occurs following *P. falciparum* – nonsterilising immunity suppressing clinical disease and rendering individuals less likely to present for treatment [8].

Individuals infected with *P. vivax* who develop clinical illness and seek medical attention often present with both asexual and sexual parasite stages in the peripheral circulation, enabling efficient transmission prior to diagnosis of the parasite and its treatment [9]. This is in marked contrast to *P. falciparum*, in which the blood stages in the peripheral circulation tend to be more synchronous, with patent gametocytaemia occurring 7–14 days after the appearance of asexual stages in acute malaria [10]. Whilst *P. vivax* gametocytes also occur after the initial treatment of the asexual infection, these are usually associated with recurrent asexual parasitaemia arising from relapse (see Glossary) of the parasite [9]. Thus, while ongoing transmission of *P. falciparum* can be reduced significantly by early diagnosis, and treatment with combination regimens – including artemisinin derivatives and a gametocytocidal agent – early treatment of *P. vivax* with this combination has less impact on transmission.

The vector biology of *P. vivax* also differs substantially from that of *P. falciparum*. *P. vivax* has evolved to survive in diverse ecological environments with varied *Anopheles* vectors. *P. vivax* gametocytes are transmitted more efficiently to *Anopheles* than are those of *P. falciparum* and are transmissible at lower parasite densities [11,12]. Parasite survival in more extreme geographical climates may be aided further by *P. vivax* sporozoites developing faster in the mosquito midgut and across a wider temperature range, compared with those of *P. falciparum* [13]. *P. falciparum* and *P. vivax* are transmitted preferentially by *Anopheles* species with different biting habits. Hence, in some coendemic locations, vector-control interventions, such as bed net distributions, may reduce *P. falciparum* malaria but have little impact on *P. vivax* transmitted by exophilic day-time vectors [14].

The defining feature of *P. vivax* is its ability to form dormant liver stages – hypnozoites that can reactivate weeks to months after an initial infection (relapse). The frequency and number of relapses vary markedly with host immunity and geographical location [15,16]. Early studies from individuals deliberately infected with malaria suggest that the number of sporozoites inoculated by the mosquito, as well as the geographical origin of parasites, are key determinants of relapse periodicity [15]. In tropical areas the risk of early relapse is high (>80%), with subsequent relapses occurring every 3–4 weeks. In temperate regions and some subtropical areas the risk of relapse is considerably lower, and there may be a long incubation or latency period between the initial illness and relapse, lasting 8–12 months.

The factors triggering the reactivation of hypnozoites are unknown, although episodic reactivation, acute febrile illness, and parasite-induced haemolysis have been postulated [15,17]. In a meta-analysis of clinical efficacy studies of patients treated for *P. falciparum* monoinfection, the greatest risk of recurrent parasitaemia was that arising from *P. vivax* rather than *P. falciparum*; overall, 24% of patients had documented *P. vivax* recurrence within 63 days [18]. The timing of recurrent vivax parasitaemia and the high efficacy of artemisinin-based combination therapies (ACTs) against the blood stages of both species, but not liver stages of *P. vivax*, suggest that the vivax recurrences were attributable to reactivation of *P vivax* hypnozoites. A subsequent individual meta-analysis shows that the greatest risk of *P. vivax* parasitaemia during follow up is in patients with delayed parasite clearance of their initial *P. falciparum* parasitaemia, consistent with either a parasite–host interaction triggering *P. vivax* relapse or vulnerability of a host with low immunity to recurrent parasitaemia [19]. Whilst there is also an increased risk of *P. vivax* in individuals following asymptomatic *P. falciparum* parasitaemia, this is considerably lower than that observed in symptomatic patients [20,21].

## *P. vivax*-Attributable Morbidity and Mortality

Historically, *P. vivax* malaria has been regarded as a benign illness; however, this dogma has been challenged as evidence of its appreciable morbidity and mortality has accumulated [22]. The aetiology of severe vivax malaria is complex, but a major contribution to morbidity is likely to be the parasite’s propensity to recur [23]. Following an acute infection, a nonimmune individual in an area of high *P. vivax* relapse periodicity can have recurrent episodes of malaria every month for over a year, a similar force of infection as that seen in areas hyperendemic for *P. falciparum* [24]. Recurrent bouts of malaria and subsequent parasite-induced haemolysis result in a cumulative risk of anaemia, particularly in young and malnourished children [25–27]. As the concentration of haemoglobin falls, the risk of mortality rises and this is compounded by concomitant infections such as pneumonia and diarrhoea [26]. In pregnancy, recurrent *P. vivax* malaria is associated with miscarriage, premature delivery, stillbirth, and low birth weight [28]. The overall direct risk of mortality in patients with acute *P. vivax* malaria has been estimated to be one in 8000 in patients presenting to community clinics – rising to one in 100 in patients admitted to hospital; however, these estimates vary markedly in different endemic settings [25,26,29,30].

Although the direct acute mortality of patients infected with *P. falciparum* is substantially greater than that of *P. vivax* [30], recent studies suggest that *P. vivax* malaria is also associated with delayed morbidity and an indirect mortality which is often ignored. In Papua, Indonesia, an area of short relapse periodicity, the risk of dying after 30 days of the initial presentation with malaria, was significantly higher in patients presenting with *P. vivax* than with *P. falciparum* [31]. Less than half of the patients who died after 30 days represented with another episode of malaria, but a high proportion were malnourished, severely anaemic, or presented with infective comorbidities such as pneumonia or diarrhoea [25,26]. These data suggest that, at least in Papua and Papua New Guinea, the actual mortality attributable to *P. vivax* has been underestimated.

## The Rising Relative Burden of *P. vivax*

**Figure 4:**
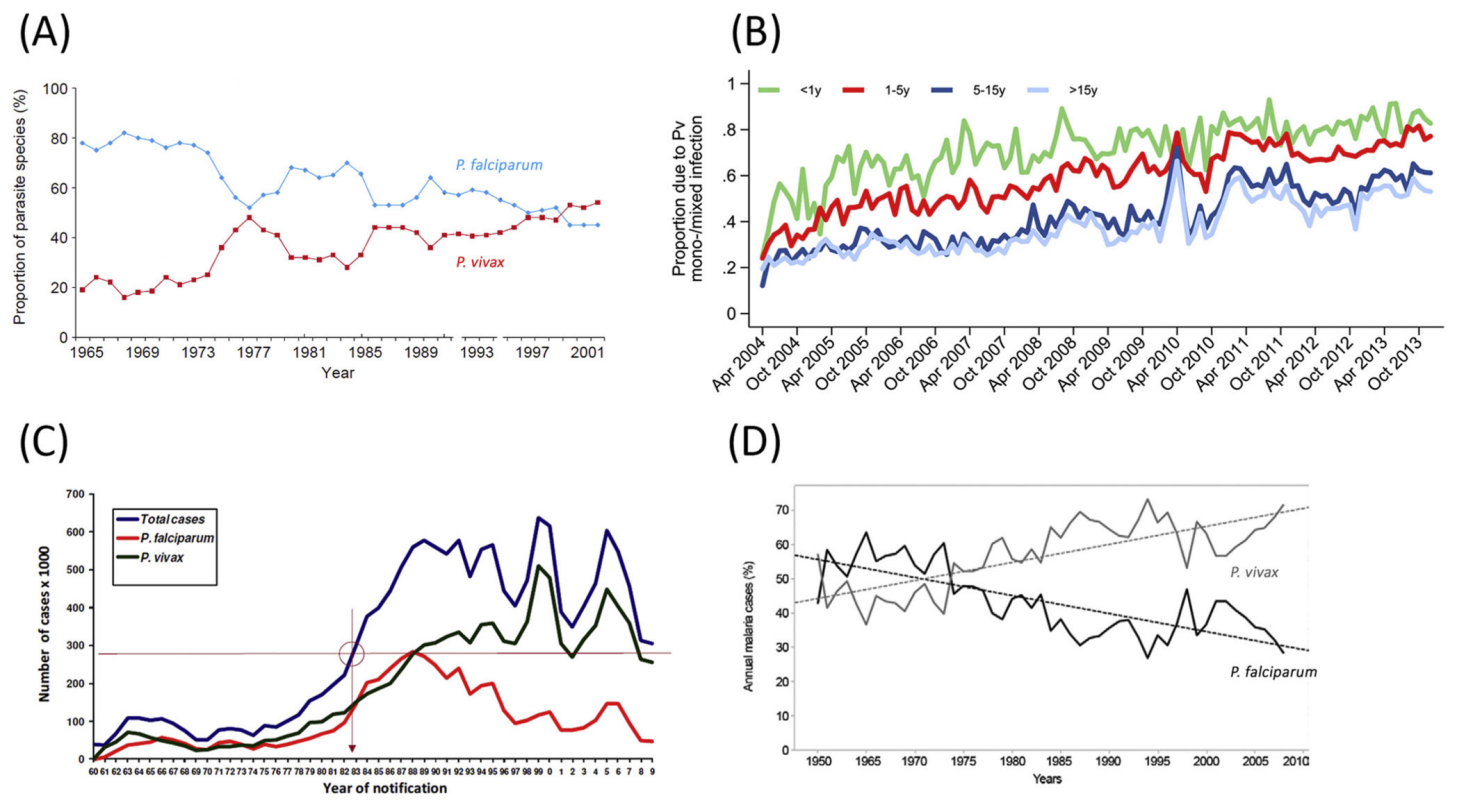
Rising Proportion of Malaria Due to *Plasmodium vivax*(Pv) Following Enhanced Malaria-Control Activities for *Plasmodium falciparum*. (A) Thailand [13], (B) Papua, Indonesia [35], (C) Brazil [33], and (D) Columbia [34].

Over the past decade, enhanced malaria-control activities, supported by increased funding for malaria-elimination activities, have led to a substantial decrease in the incidence of malaria in many malaria-endemic countries [32]. However, there has been a consistent increase in the proportion of malaria due to *P. vivax* (Figure 4) [13,33–35]. This increase is likely due to multiple factors, including reporting practices, the ability to detect and treat infected individuals effectively, greater resilience of *P. vivax* to standard malaria-control measures, and the parasite’s transmission dynamics (Table 1).

In 2006, Indonesia became the first malaria-endemic country to adopt a universal policy of an ACT for the treatment of malaria due to any species. Following the implementation of dihydroartemisinin-piperaquine in the western province of Papua, the incidence of malaria fell by more than a half, and this was associated with a significant fall in the proportion of malaria requiring admission to hospital and malaria-related mortality. The impact of ACT on *P. vivax* was less than that on *P. falciparum*, with the proportion of malaria attributable to *P. vivax* rising from 20% to 52%; in this region, 80% of malaria in young children is now due to *P. vivax* [35]. In nearby Papua New Guinea, where *P. vivax* is also the predominant cause of malaria in children, enhanced vector control, and a universal policy of artemether-lumefantrine for uncomplicated malaria, resulted in a marked decline in clinical malaria due to both falciparum and vivax, although the prevalence of *P. vivax* and asymptomatic infection remained high long after the early reductions in *P. falciparum* malaria [36]. In Thailand, Chu *et al*. reported that increased access to early diagnosis and treatment was associated with an early decline in *P. falciparum* infection – but a delayed fall in *P. vivax* malaria; the authors hypothesize that the reduction in *P. falciparum* transmission may have resulted in reduction of *P. vivax* infections due to decreasing relapse activation [37].

Over 80% of *P. vivax* malaria cases in the Asia Pacific region are estimated to arise from reactivation of dormant liver stages and clinical relapse [38,39]. Hence, the hypnozoite reservoir constitutes a major preventable burden of malaria that must be addressed to eliminate the parasite [40]. In most countries where malaria has been eliminated, the transmission of *P. falciparum* was interrupted several years before that of *P. vivax*. In Sri Lanka and Malaysia the falling burden of malaria allowed greater attention to be given to ensuring widespread access to effective radical cure, and patients treated with a 14 day regimen of primaquine could be supervised directly to monitor safety and encourage full adherence [41,42]. Whilst enhanced control efforts that do not include radical cure can reduce clinical cases considerably, they need to be sustained for prolonged periods to achieve elimination [37].

## *P. vivax* Radical Cure

The radical cure of patients with vivax malaria requires treatment with a combination of both schizonticidal and hypnozoiticidal antimalarial drugs. The former clears the peripheral blood stages of the parasite to achieve resolution of the acute febrile illness and the latter kills the hypnozoite reservoir, preventing subsequent relapsing infections and onward transmission.

In most countries, chloroquine remains the mainstay of treatment for *P. vivax* blood stages. However, chloroquine-resistant (CQR) *P. vivax* has emerged and is now apparent in many areas, although the degree to which clinical efficacy is compromised varies considerably [43]. High-grade chloroquine resistance is apparent only on the island of Papua (Indonesia and Papua New Guinea), where there is intense transmission, and Sabah (Malaysia) which is in the final stages of elimination [44–46]. Elsewhere, CQR is mostly low grade and may even be transient, potentially reflecting the challenges of defining drug resistance in *P. vivax*, fitness disadvantages arising from the molecular acquisition of CQR, or the parasite’s transmission dynamics [47,48].

The use of suboptimal chloroquine dosing also increases the risk of recurrent *P. vivax* [49]. In a meta-analysis of individual pooled patient data, increasing the total dose of chloroquine from 25 mg/kg to 30 mg/kg, was predicted to reduce the number of recurrent infections by 41% in young children [50]. More importantly, however, was combining chloroquine with primaquine radical cure which reduced early recurrent *P. vivax* parasitaemia by 90%, likely through a combination of primaquine’s synergistic activity with chloroquine against the asexual blood-stage parasites and its hypnozoiticidal activity preventing early relapses [50]. To combat the declining susceptibility of *P. vivax* to chloroquine, five countries have adopted a policy of universal ACT for both *P. falciparum* and *P. vivax*: Indonesia, Papua New Guinea, Solomon Islands, Vanuatu, and Cambodia. ACTs, with the exception of artesunate-sulfadoxine-pyrimethamine, generally have excellent efficacy against CQR *P. vivax*, and a unified ACT-based treatment protocol has operational efficiencies and significant pragmatic advantages [51]. Comparison of the short-term efficacy of different ACTs against *P. vivax* highlights the benefits of combinations with a long-acting partner drug, such as artesunate-mefloquine or dihydroartemisinin-piperaquine, which have 90% fewer recurrences at day 42 compared with patients treated with the shorter-acting artemether-lumefantrine [52]. Suppression of the first relapse delays the time to symptomatic recurrence, and this is associated with improved haematological recovery [52].

Primaquine, an 8-aminoquinoline compound, is the only widely available hypnozoiticide capable of killing *P. vivax* hypnozoites and thereby preventing relapses. However, 8-aminoquinoline drugs can cause severe drug-induced haemolysis in individuals with glucose-6-phosphate dehydrogenase (G6PD) deficiency [24]. In view of the risk of haemolysis to the foetus and newborn infant, primaquine is contraindicated in pregnant women and lactating mothers. Although a recent study suggests that there is minimal excretion of primaquine in breast milk, treatment guidelines have yet to be revised to recommend primaquine use in lactating women [53].

The degree of primaquine-induced haemolysis depends upon the erythrocyte G6PD activity of the individual exposed. Up to 30% of patients in malaria-endemic communities have this X-linked enzyme deficiency, with more than 180 different G6PD deficiency alleles reported. Although the absolute haemolytic risks of different variants and the relationship with enzyme activity are largely unknown [54], there is a clear relationship between haemolysis and daily and total dose of primaquine administration [24].

WHO recommends routine testing of G6PD deficiency prior to primaquine administration; however, in poorly resourced communities this is rarely possible, and hence radical cure is usually prescribed without prior G6PD testing. To mitigate the risks of drug-induced haemolysis, many countries recommend a total primaquine dose of 3.5 mg/kg (15 mg per day in adults) spread over 14 days. The WHO recommends a higher dose (total 7 mg/kg or 30 mg per day in adults), also spread over 14 days, in areas where frequent-relapsing *P. vivax* is prevalent, such as in East Asia and Oceania [55]. In routine clinical practice, daily supervision is rarely possible and the prolonged treatment regimen is associated with poor adherence and effectiveness [56,57].

The antirelapse efficacy of primaquine is determined by the total dose of drug administered [24]. A systematic review in 2012 identified 18 studies in which schizonticidal treatment with primaquine was compared with a schizonticidal treatment alone [58]. In studies administering a low total dose of primaquine (2.5–5.0 mg/kg), primaquine reduced *P. vivax* recurrences sevenfold, but in studies administering a high-dose regimen (>5.0 mg/kg) the risk of recurrence was reduced by 33-fold. Only two randomized controlled trials, with follow up to 6 months, have compared 14 days of high- versus low-dose primaquine – both were conducted in India where the risk of relapse is generally low [59,60]. The combined relative risk at 6 months was 0.82, although the 95% confidence intervals crossed parity [61]. These meta-analyses are confounded by comparison of trials with different durations of follow up, marked heterogeneity in the risk and timing of relapse in different locations, differing endpoints, and small sample sizes.

Two recent clinical trials have demonstrated that a 7-day high-dose regimen has similar efficacy to the same total dose administered over 14 days [62,63], the risk of recurrent *P. vivax* malaria at 12 months ranging from 7% to 20% across nine sites located in Afghanistan, Ethiopia, Indonesia, Thailand, and Vietnam. Whilst shortening the treatment course of primaquine has potential to improve adherence, this requires a higher daily dose which increases the risk of haemolysis and gastrointestinal intolerance. Although these adverse effects can be reduced by point-of-care G6PD diagnostics and administration of the drug with food, the tolerability and effectiveness of high-dose 7-day primaquine has yet to be determined in clinical practice. Host factors also play an important role in antirelapse efficacy, including the ability of patients to convert the primaquine into its active metabolites. Primaquine is metabolized in the liver by monoamine oxide (MAO) and cytochrome P450 (CYP450) enzymes (notably the 2D6 isotype – CYP2D6) [64]. CYP2D6 is naturally polymorphic, and some variants, present in up to 25% of the population, are associated with significant reductions in antirelapse efficacy [65,66].

In 2018 tafenoquine, an 8-aminoquinoline drug with a longer terminal elimination half-life than primaquine, was registered with the FDA and the Australian Therapeutic Goods Administration (TGA) [67]. When administered with chloroquine, a single dose of 300 mg of tafenoquine has similar efficacy to a 14-day low-dose (3.5 mg/kg total dose) primaquine regimen – the risk of recurrent *P. vivax* malaria at 12 months ranging from 31% to 41% across eight sites located in Ethiopia, Peru, Brazil, Cambodia, Thailand, and the Philippines [68,69]. Tafenoquine is currently not licenced in children, although clinical trials are ongoing. Tafenoquine’s slow elimination from the peripheral circulation raises concerns of sustained drug-induced haemolysis, and this has led to it being recommended only in patients with a G6PD enzyme activity of 70% or greater. Diagnosis of G6PD deficiency at this level of enzyme activity requires the use of a quantitative G6PD assay [70,71]. By contrast, primaquine is currently recommended for patients with a G6PD enzyme activity as low as 30%. In the past decade, novel point-of-care G6PD tests have been developed, bringing a new era in which the widespread use of novel 8-aminoquinoline regimens, such as tafenoquine and/or short-course high-dose primaquine, can be explored and implemented [72,73].

## Universal Radical Cure

The high risk of *P. vivax* parasitaemia following *P. falciparum* infection suggests that, in some coendemic areas, a universal policy of radical cure for patients with uncomplicated malaria due to either *P. vivax* or *P. falciparum* may offer significant benefits both for the individual and the community. Such a strategy would include an ACT to treat the asexual stages of all *Plasmodium* species plus a hypnozoiticidal agent (either primaquine or tafenoquine) to eradicate the liver stages of *P. vivax* and *Plasmodium ovale*. Preventing recurrent parasitaemia can reduce acute morbidity and mortality directly associated with malaria and indirectly from diseases associated with a cumulative impact of repeated episodes of malaria [31]. At a community level a universal policy would also prevent the ongoing transmission of *P. vivax* and the infection of others [40].

Universal radical cure has been used in a cohort of children in Papua New Guinea, resulting in 90% reduction in *P. vivax* parasitaemia at 1 year compared with those treated with placebo [38]. On the Thailand–Myanmar border the radical cure of 12 individuals with asymptomatic *P. falciparum* was predicted to prevent one subsequent infection with *P. vivax* [20]. However, the risks and benefits of this strategy will vary considerably between endemic settings and over time. Indeed, on the Thailand–Myanmar border the risk of vivax following falciparum fell significantly as the prevalence of *P. vivax* decreased [74]. Prospective clinical trials are in progress.

## Concluding Remarks

Although the global burden of *P. vivax* is considerably lower than that of *P. falciparum* it remains prevalent across most of the malaria-endemic world, exerting a considerable public health burden and cost to both infected individuals and communities. Outside of sub-Saharan Africa, *P. vivax* is becoming the predominant cause of malaria since intense malaria-control interventions, which prioritize the control of *P. falciparum*, generally have less impact on reducing the transmission of *P. vivax*. As the global community strives to achieve the elimination of malaria from all endemic countries, it is imperative that radical cure of the *P. vivax* parasite be implemented widely if the proposed ambitious timelines are to be met. Despite 60 years of widespread use, key questions regarding optimal strategies for radical cure remain (see Outstanding Questions). Policy makers and healthcare providers have to address these as a matter of urgency and find ways of providing radical cure to children and patients in remote rural areas – where most vivax malaria occurs. The recent development of novel diagnostic tools and treatment strategies provides fresh impetus to deliver radical cure safely and effectively. In view of the inherent haemolytic risks of treatment with 8-aminoquinoline drugs, an integrated package of interventions will be required, including patient education, community engagement, consistent supply chains, and robust health systems, with sufficient funding to maintain these until the ultimate elimination of the parasite has been achieved.

## Figures and Tables

**Table 1:** Reasons why Conventional *Plasmodium falciparum* Malaria-Control Activities have less impact on the Burden of Disease of *Plasmodium vivax*, resulting in an increasing Proportion of Malaria being attributable to *P. vivax*

Transmission dynamics		Refs
	Appearance of sexual-stage parasites prior to clinical presentation and start of antimalarial treatment	[9]
	A high proportion of recurrent infections attributable to relapse, which are associated with transmissible gametocytaemia	[9]
	Efficient transmission of *P. vivax* to mosquito at low-level parasitaemia	[12,13]
	Exophilic and daytime biting vectors may reduce the impact of bed net distribution and indoor residual spraying	[14]
Reduced Detection of *P. vivax*		
	Low sensitivity of rapid diagnostic tests to detect peripheral *P. vivax* parasitaemia	[7]
	Rapid acquisition of immunity results in a high proportion of asymptomatic carriers, that goes undetected and untreated	[8]
	High proportion of infected individuals with asymptomatic hypnozoite parasites	[15]
	Mixed-species infections misreported as *P. falciparum* monoinfection	[75]
	Increasing recognition and reporting of different *Plasmodium* species causing malaria, rather than assumption that malaria is due to *P. falciparum*	
Ineffective treatment of *P. vivax*		
	Healthcare providers prioritize the treatment of the acute febrile illness rather than the prevention of subsequent relapses	[76]
	Poor adherence to a 14-day radical cure treatment regimen	[57]
	Poor access to rapid and reliable G6PD tests prevents identifying patients at risk of primaquine-induced severe haemolysis	[76]
	Sub-optimal (lower total dose) primaquine regimens being recommended, due to concerns about severe haemolytic reactions	[58]
	CYP2d6 polymorphisms reduce the primaquine metabolism and hypnozoiticidal efficacy	[65]

## Highlights

*Plasmodium vivax* infection is present across most of the malaria-endemic world.

*P. vivax* relapses, arising from dormant liver stages, cause recurrent episodes of malaria that are the main determinant of significant morbidity, mortality, and ongoing transmission.

In coendemic areas, where intense malaria-control activities have reduced the burden of *P. falciparum*, there has been a rise in the proportion of malaria attributable to *P. vivax*.

There is an increased risk of *P. vivax* after the treatment of *P. falciparum* in coendemic regions, suggesting potential benefit of universal radical cure for both parasites in some areas.

Novel host and parasite diagnostics, single-dose tafenoquine, and short-course primaquine regimens offer hope for safer and more effective radical cure to eliminate the parasite.

## Glossary

**Artemisinin-based combination therapy (ACT)**: an antimalarial drug regimen that includes an artemisinin derivative combined with a longer acting antimalarial treatment; it is now globally recommended for the treatment of *P. falciparum* infection and for chloroquine-resistant *P. vivax*.

**CYP2D6**: an isotype of the cytochrome 450 enzyme involved in primaquine metabolism. Polymorphisms are associated with reduced primaquine efficacy.

**Glucose-6-phosphate dehydrogenase (G6PD) deficiency**: an inherited enzymopathy that makes the host vulnerable to oxidative stress and severe haemolysis following administration of primaquine or tafenoquine.

**Hypnozoites**: the dormant liver stages of *P. vivax* and *P. ovale* that can reactivate and relapse weeks to months after the initial infection.

**Radical cure**: the treatment and eradication of all parasite stages in the host, including the asexual parasites associated with symptoms, the sexual stages causing transmission to the mosquito vector, and the dormant liver stages (hypnozoites) that can cause relapsing infection. It requires a combination of schizonticidal drug (s) plus a hypnozoiticidal agent (primaquine or tafenoquine).

**Recurrent parasitaemia**: a repeat episode of peripheral asexual parasitaemia following initial treatment of malaria.

**Relapse**: recurrent parasitaemia arising weeks or months after an initial infection of *P. vivax* or *P. ovale*, following reactivation of the dormant liver stages (hypnozoites) that generate a new blood-stage infection.

**Universal radical cure**: the use of a radical cure in patients with uncomplicated malaria irrespective of the *Plasmodium* species causing the peripheral parasitaemia.

## Outstanding Questions

What factors determine the activation of *P. vivax* relapse, and how do the timing and frequency of relapses differ across endemic settings?

What is the optimal dose of primaquine needed to achieve high antirelapse efficacy in different endemic regions?

Is a short-course, high-daily dose of primaquine (7 days or even shorter) more effective in clinical practice than a 14-day regimen, and what measures can be taken to ensure patient adherence to a full treatment course?

Is a single 300 mg dose of tafenoquine as efficacious and effective as a high-dose primaquine regimen (7 mg/kg total), and does it retain its efficacy when combined with an artemisinin-based combination therapy?

Where and how can qualitative or quantitative G6PD testing be used reliably and cost-effectively in routine clinical practice, and, in areas where their use is not feasible or affordable, how can radical cure be administered safely?

What measures should be taken to detect early signs of haemolysis and mitigate subsequent clinical sequelae in vulnerable individuals?

Can a universal policy of radical cure for both *P. viva*x and *P. falciparum* reduce the risk of parasitaemia following uncomplicated malaria, and where would such a policy be most beneficial?

Can rapid diagnostic tests for *P. vivax* be improved to a level at which they can replace blood film microscopy in elimination programmes?

How can community engagement be used to inform patients and healthcare providers of the nature of *P. vivax* malaria and the risks and benefits of radical cure?

What are the optimal combinations of vector control and radical cure interventions required to fast-track *P. vivax* elimination in different endemic and resource settings?

What are the key operational challenges for national malaria programs when implementing radical cure, and how can these be overcome?
